# Iodine: a scoping review for Nordic Nutrition Recommendations 2023

**DOI:** 10.29219/fnr.v67.10369

**Published:** 2023-12-26

**Authors:** Ingibjörg Gunnarsdóttir, Anne Lise Brantsæter

**Affiliations:** 1 Faculty of Food Science and Nutrition, University of Iceland, Reykjavik, Iceland; 2 Unit for Nutrition Research, Landspitali National University Hospital, Reykjavik, Iceland; 3 Department of Food Safety, Division of Climate and Environmental Health, Norwegian Institute of Public Health, Oslo, Norway

**Keywords:** iodine, thyroid function, goitre, nutrition recommendations

## Abstract

Iodine is essential for the synthesis of the thyroid hormones thyroxine (T4) and triiodothyronine (T3). As in many other parts of the world, insufficient iodine intake and consequently insufficient iodine status is a public health challenge in the Nordic and Baltic countries. The main dietary sources of iodine in the Nordic and Baltic countries include cow’s milk, saltwater fish, eggs, products containing iodised salt, and iodised table salt. Only Denmark (DK), Finland (FI) and Sweden (SE) have implemented mandatory (DK) or voluntary (SE, FI) salt iodisation. New data, as well as recent studies from the Nordic and Baltic countries, strengthen the evidence that the main health challenges related to insufficient iodine intake remain thyroid function and thyroid disease, mental development, and cognitive function. Excessive intakes can also cause hyperthyroidism, autoimmune thyroid disease, and thyroid cancer.

## Popular scientific summary

- Iodine is an essential nutrient for synthesis of thyroid hormones and a normal thyroid function in humans.
- Insufficient intake of iodine can lead to reduced thyroid function, mental development, and cognitive function.
- Cow’s milk, saltwater fish, eggs, and iodised salt are the main sources of iodine in the Nordic and Baltic diets.
- Iodine deficiency is also a public health issue in the Nordic and Baltic countries.

Iodine is an essential component for the synthesis of the thyroid hormones thyroxine (T4, a pro-hormone) and triiodothyronine (T3, the active hormone). During the foetal stage, infancy and childhood, these hormones are crucial for growth and numerous processes of neural and cognitive development, e.g. myelinisation, neural migration and differentiation, and gene expression (1–4). Although iodine excess is not considered a large-scale public health problem, excessive intake can also have negative effects on thyroid function in vulnerable individuals including foetuses and infants. The relationship between iodine and thyroid disease in a population is U-shaped, with an increased risk with both low and high iodine intake (5, 6). The most recognisable consequence of iodine deficiency and iodine excess is enlargement of the thyroid gland. Other consequences include hypothyroidism, decreased fertility, adverse pregnancy and birth outcomes, and impaired neurocognitive development in children (7, 8). Considerable progress has been made in prevention and control in recent decades due to successful monitoring and salt iodisation (9, 10). Nevertheless, iodine deficiency continues to be the most common cause for preventable brain damage worldwide (11). Universal salt iodisation is the recommended intervention for preventing and correcting iodine deficiency (12). The World Health Organisation (WHO) recommendation for universal salt iodisation is based on a comprehensive systematic review (SR) and meta-analysis showing that iodised salt reduces the risk of a wide array of adverse health outcomes caused by iodine deficiency (13). However, the WHO guideline for implementation of salt iodisation has only been applied in some of the Nordic and Baltic countries, as country specific food patterns differ, and some countries have a history of being iodine sufficient due to high consumption of milk and fish. In 2000, mandatory iodisation of table salt and bread salt was introduced in Denmark as a response to studies showing low iodine status and thyroid abnormalities in adult population groups (14, 15). In 2004–2005, urinary iodine excretion had increased significantly in all age groups compared with the excretion levels before mandatory iodine fortification (16). Before 1950, there was endemic iodine deficiency in Norway, Sweden and Finland (17, 18). Iodine fortification of cow fodder resulted in a relatively high concentration of iodine in milk and dairy products, and high levels of consumption of these products led to eradication of endemic goitre (increased thyroid gland size) in Norway (19). In Finland, it was both the addition of iodine to cow fodder and the introduction of iodised table salt that erased endemic goitre (18). Iceland was for a long time known for its high iodine status, which was likely due to high levels of fish consumption (20). With time, dietary patterns have changed in all Nordic and Baltic towards decreased intakes of milk and white fish. Insufficient iodine intake is still a public health challenge in the Nordic and Baltic countries because of insufficient or lacking legislation on universal salt iodisation (18).

## Methods

This scoping review of new literature regarding iodine followed the protocol developed within the Nordic Nutrition Recommendations 2023 (NNR2023) project (21) (Box 1). The sources of evidence used in the scoping review follow the eligibility criteria described previously (22).

Box 1 Background articles for Nordic Nutrition Recommendations 2023

- This article is one of many scoping reviews commissioned as part of the Nordic Nutrition Recommendations 2023 (NNR2023) project (21)
- The articles are included in the extended NNR2023 report but, for transparency, these scoping reviews are also published in Food & Nutrition Research
- The scoping reviews have been peer reviewed by independent experts in the research field according to the standard procedures of the journal.
- The scoping reviews have also been subjected to public consultations (see report to be published by the NNR2023 project).
- The NNR2023 committee has served as the editorial board.
- While these articles are a main fundament, the NNR2023 committee has the sole responsibility for setting dietary reference values in the NNR2023 project.

Qualified and relevant SRs are the main evidence-based documentations used while setting dietary reference values (DRVs) in NNR2023. An initial scoping review for iodine was carried out by the NNR2023 Committee in accordance with the PRISMA guidelines (22), to evaluate if topics relevant to iodine should be shortlisted for a new SR within the NNR2023 project. The initial search was first conducted in PubMed on January 11th, 2020, with the following search string: *iodine [MeSH Terms] AND (‘2011’[Date – Publication]: ‘3000’[Date – Publication]) AND Humans [Filter] AND (‘Diet’ OR ‘Dietary’ OR ‘Food’ OR ‘Nutrition’ OR ‘Nutritional’) AND systematic review [Publication Type].* This retrieved several high-quality SRs (1, 13, 23–28), leading to the conclusion that no subtopic of iodine should be shortlisted for a *de novo* SR in the NNR2023 project (29).

The main literature search for the present scoping review was performed on May 12th, 2021, where three additional SRs found to be relevant for the NNR were retrieved using the same search string as in the initial search (30–32). An updated search was performed on April 4th, 2022, identifying one new SR relevant for the NNR (33). Furthermore, three additional SRs were retrieved from the initial search at this stage (34–36). A recent randomised, double-blind, dose-response crossover iodine balance study, with low risk of bias, was used when suggesting change in the DRV for infants and children below 2 years (37).

## Physiology

Dietary iodine is in general rapidly and efficiently (>90%) absorbed in the small intestine as inorganic iodide (I-) (38, 39). Iodine is then actively transported from the circulation and concentrated in the thyroid (38, 40–42). The rate of clearance from circulation depends on iodine intake, and is 10% or less in sufficiency, but can reach 80% in chronic deficiency. The thyroid stores up to 80% of total body stores, reaching up to 20 mg in healthy adults, but 300 µg in infants (39, 43, 44). When iodine intake is sufficient, the kidneys excrete >90% of ingested iodide in urine as iodide (38) in a process involving both glomerular filtration and tubular reabsorption. Small amounts of iodide are lost through skin and faeces (41, 42, 45). Iodine absorption and utilisation can be affected by goitrogens, mainly sulphur-containing glucosides (glucosinolates). These are dietary constituents that can inhibit the uptake of iodine into the thyroid gland (e.g. thiocyanates) or interact with hormone production (e.g. goitrins) (46). These compounds occur in Brassica species such as cabbage, Brussels sprouts, turnips, and rapeseeds. The levels of glucosinolates in most diets are generally too low to have an impact on iodine status (47). Goitrogenic effects of thiocyanate from cigarette smoking is also well known (48).

During pregnancy iodine requirements increase to fulfil foetal and maternal thyroid hormone needs (49). Three main factors contributing to increased maternal iodine needs include: (1) approximately 50% increase in maternal thyroid hormone production, (2) increased iodine clearance by kidneys, (3) transfer of maternal iodine to foetus via the placenta (50, 51).

Trans-placental iodine transfer and increased renal iodine clearance of 30–50% contributes to increased iodine requirements (51). In the early stages of intrauterine life, organogenesis is nearly complete by the 12th week of gestation; therefore, thyroid hormone insufficiency during the first trimester of pregnancy may irreversibly affect the neurodevelopment of the progeny (52). From 16 to 20 weeks of gestation, the foetal thyroid becomes active, but remains solely dependent on maternal iodine supply for proper functioning and thyroid hormone production (53). As a result, maternal iodine stores are crucial to sustain levels of thyroid hormones needed for successful foetal development and pregnancy outcomes (3, 54).

Infancy is a period of faster growth than any other period in life, and due to its importance for growth, thyroid hormone production is at a rate of 5–6 μg/kg body weight/day in infancy, but drops to 1.5 μg/kg body weight/day in healthy adults. The gland has limited storage capacity at this early age, and is not able to increase fractional clearance to the same extent as the adult thyroid does during deficiency. In accordance with that, the thyroid requires more iodine per kg of body weight in infancy than in other periods of life (37, 55, 56). This is evident in areas of deficiency where infants are more susceptible to hypothyroidism than their lactating mothers, pregnant women, and women of reproductive age (56, 57).

Iodine is secreted into breastmilk at a concertation gradient 20 to 50 times that of plasma through increased expression of the sodium/iodide symporter present in breast cells (58). It has been estimated that 40–45% of maternal consumption is excreted into breast milk. Thiocyanate from smoking may inhibit the iodine transport in the mammary gland and reduce iodine content of breastmilk (59).

Breast milk iodine concentrations of around 150 µg of iodine per litre has been reported from areas where salt iodisation programmes have been implemented, while the content is much lower in areas with high prevalence of goitre (9–32 µg/L) (60, 61). No reference range has been specified for breast milk iodine concentration, but studies suggest that positive iodine balance of full-term infants is reached at breast milk iodine concentration of 100–200 µg/L (60, 61). When iodine intake is inadequate, compensatory mechanisms enhance iodine transport to breastmilk, but may not be adequate to ensure sufficient iodine intake in breastfed infants (58). Iodine concentration in breastmilk samples from the Nordic countries range from median concentrations of 68–71 μg/L in Norway (62, 63), 84 μg/L in Iceland (64), 90 μg/L in Sweden (65), and 83 μg/L in Denmark (66), suggesting that breast milk iodine concentration may not be sufficient to meet the iodine requirement for breastfed infants.

## Assessment of iodine status

Several complementary indicators are used for assessment of iodine status; urinary iodine concentration (UIC), thyroid volume (TV), serum thyroid stimulating hormone (TSH), thyroid hormones, and serum thyroglobulin (Tg). Median UIC in spot urine samples is the recommended indicator to assess iodine status in populations (67). UIC is a good marker of short-term iodine status (i.e. days), and although UIC at the individual level varies with recent food intake and hydration status, the median UIC is a valid marker of iodine intake at the group level (10, 39). UIC cannot be used to determine the proportion of the population with iodine deficiency or excess. However, having two independent spot samples from a subsample of the study population can be used to estimate the habitual long-term iodine intake and the prevalence of deficiency and excess (8). In school aged children and non-pregnant adults, iodine intake is considered sufficient when the median UIC in the population is 100–299 µg/L (67). In pregnant women, iodine intake is considered sufficient when the median UIC is 150–249 µg/L (68).

Daily iodine intake for population estimates can be extrapolated from UIC, using estimates of mean 24-h urine volume using the equation: UIC (µg/L) × 0.0235 × body weight (kg) = iodine intake (µg/day) (69), assuming 90% excretion and 1.5 litre urine per 24 h. Thus, a median UIC of 100 µg/L in an adult corresponds roughly to an average daily intake of 150 µg. The approach does not account for iodine uptake in the thyroid and is less valid in iodine-deficient situations and during pregnancy and lactation.

The median cut-off of UIC at ≥100 µg/L as an indicator of sufficient iodine intake was established for school aged children, and there is an ongoing debate whether this cut-off is too high for non-pregnant adults (10). The WHO cut-off for median UIC in pregnant women at 150 µg/L corresponds to an intake of 250 µg/day, while the previous Nordic recommendation from 2012 for iodine intake of 175 µg/day (70) corresponds to a median UIC of 105 µg/L, which is closer to the cut-off for non-pregnant adults.

Other biomarkers include TSH and the thyroid hormones T4 and T3. However, the normal reference ranges are wide, and these biomarkers are useful only in moderate to severe iodine-deficiency. Tg is a precursor of thyroid hormones and is a longer-term biomarker of iodine status compared to UIC for monitoring iodine status in children and adults. Elevated Tg has been proposed to be a sensitive biomarker of both iodine deficiency and excess, but validated cut-offs in adults are lacking (71, 72).

Goitre prevalence has been used as measure of iodine deficiency, but TV measurement is a reliable indicator of goitre prevalence only in areas of moderate and severe deficiency and not in areas with milder iodine deficiency (71, 73).

## Iodine intake in Nordic and Baltic countries

Ideally, iodine status should be assessed by biomarkers, but rough estimates of iodine status can also be made by dietary assessment using multiple diet records, repeated 24-h recalls or food frequency questionnaires (FFQ), comparing the estimated habitual iodine intake with DRVs. While precision is a limitation of all dietary assessment methods, assessing dietary iodine intake is even more demanding. This is because quantification of iodine from iodized salt both at the table and in cooking makes dietary assessment particularly difficult. Iodised salt in food production can vary, if it is practised in a country, and there is also a considerable variation in the iodine concentrations in foods, such as fish and dairy.

Iodine intake estimated from food and dietary supplements should be validated by assessing urinary iodine in a subsample of participants (71). A large variation in iodine intake can be seen in the Nordic and Baltic countries, both between countries as well as gender and age groups. The main sources of iodine include cow’s milk, saltwater fish, eggs, iodized table salt and products containing iodized salt, such as bread (18, 74). The lowest iodine intake is reported in Lithuania (with mean intake around 30 µg/d for adult men and women) (74). However, intake of iodine from supplements or salt was not included in the estimate. Iodine intake in other countries range from an average of 94 µg/d in adult women in Latvia to 268 µg/d for adult men in Denmark (74).

## Health outcomes relevant for Nordic and Baltic countries

Iodine deficiency remains a public health problem in many subgroups and regions around the world, including the Nordic and Baltic countries (64, 73, 75–81). The main health-related challenges include thyroid function and thyroid disease, mental development and cognitive function, and excessive iodine intake.

### Thyroid function and thyroid disease

The role of iodine in chronic diseases is primarily through thyroid dysfunction. Worldwide, iodine deficiency is the main cause of thyroid disorders, including hypothyroidism (82). However, chronic exposure to excess iodine may also cause hypothyroidism (24). A SR and meta-analysis of iodine intake and thyroid diseases concluded that the prevalence of most thyroid diseases is lowest in populations with median UIC in the range 100–299 µg/L (27). Data from Denmark have shown that hypothyroidism prevalence decreases in populations with mild iodine deficiency compared to those with severe deficiency, while autoimmune hypothyroidism prevalence increases as population iodine intake increases to sufficiency or excess (83). The clinical implications of hypothyroidism relate to nearly all organs and affect both physical and mental health, e.g. metabolic, cardiovascular and neurocognitive disorders (84). Furthermore, both low and high iodine intakes may contribute to the development of thyroid cancer (25, 85), while the overall incidence of thyroid cancer is not influenced by iodine intakes within the normal range from dietary sources. Data from countries before and after implementation of salt iodisation have shown a change in the distribution to less malignant subtypes and decrease in thyroid cancer mortality (85, 86). In mild-to-moderate iodine deficiency, increased thyroid activity can compensate for low iodine intake and maintain normal thyroid function in most individuals, although chronic thyroid stimulation will result in an increase in the prevalence of toxic nodular goitre and hyperthyroidism in populations (7). Furthermore, some studies indicate that an abrupt increase in iodine intake, e.g. initiation of iodine supplement use in pregnancy, may result in a transient stunning effect on the thyroid gland, inhibiting the release of thyroid hormones (50, 87).

### Mental development and neurocognitive function

Iodine deficiency has been described as the single greatest cause of preventable mental impairment (39). In areas with chronic moderate to severe iodine deficiency, children score an estimated 7–10 points lower on IQ tests (1, 13). Iodine deficiency can present as a spectrum of disorders depending on the degree of severity. In regions of severe iodine deficiency, i.e. median UIC < 20 µg/L in school-aged children and adults (67), adverse physical and mental health effects are well documented, and there is convincing evidence that iodine supplementation initiated prior to or in early pregnancy improves child physical and mental development (7, 39). In regions of mild-to-moderate iodine deficiency, i.e. median UIC in pregnant women <150 µg/L (67), the evidence for adverse effects is limited, suggestive (28, 49, 88). This also applies to studies on iodine supplementation in pregnancy (30, 32). Adverse effects associated with mild-to-moderate iodine deficiency are more consistent when maternal median UIC is below <100 µg/L (cut-off in non-pregnant adults) than when median UIC is higher than 100 µg/L but below <150 µg/L (cut-off for pregnant women), as for example lower cognitive scores and poorer school performance in children reported in European pregnancy cohorts (89–94). Furthermore, there is no clear evidence of beneficial effects of maternal iodine supplementation on child neurodevelopmental outcomes in pregnant populations with median UIC in the range 100–150 g/L (35, 95, 96).

### Excessive intake

Excessive intakes can cause hyperthyroidism, autoimmune thyroid disease, and thyroid cancer (25). Goitre caused by excessive intakes is prevalent in populations that reside in coastal regions and consume seaweed, like Japan. Other countries have excessive intakes as a result of iodine-rich drinking water (China) or excessively iodised salt (Brazil, Georgia) (24, 49). Subgroups in populations with adequate intakes can also be exposed to excessive iodine through iodinated contrast media, iodine-containing antiseptics, supplements or natural products (6). In the Nordic countries there is an increasing interest in seaweed as a resource for future nutrition. Algae are often suggested as a vegetarian source of iodine, especially in vegan diets. However, because the content of iodine in algae vary considerably (some varieties might contain toxic amounts), the iodine content of algae should be known and consumers should be aware of the risk of excess iodine intake (97, 98).

## Requirement and recommended intakes

### Adults and adolescents

The recommended intake (RI) in NNR2012 for adults and adolescents was set to 150 µg/day (70). The RI for adults was equal to that by WHO, the European Food Safety Authority (EFSA) and the US Institute of Medicine (IOM, now National Academy of Sciences, Engineering, and Medicine), while the NNR2012 recommendation for adolescents was slightly higher than those by EFSA and IOM (67, 69, 99). The requirements for iodine were based on thyroid iodine accumulation and turnover. The iodine requirement to prevent goitre is estimated to be 50–75 µg/day or a daily intake of approximately 1 µg/kg bodyweight (67, 69, 99–101). The average requirement (AR) was estimated to be 100 µg/day for both adult women and men, and at this intake the iodine concentration in the thyroid gland reaches a plateau. The daily iodine turnover in subjects with normal thyroid function is at a similar level (69). The RI of 150 µg/day for adults and adolescents includes a safety margin for any goitrogenic substances. The lower limit of intake for adults was estimated at 70 µg/day.

### Infants and children

In NNR2012, the RI for infants and children were based on data on goitre prevalence and urinary iodine excretion in European children and on extrapolations from adults based on energy and growth requirements (about 1–2 µg/kg body weight plus a 100% safety margin) (102). An intake of 50–70 µg/day was estimated to be sufficient for infants below the age of 2 years, assuming iodine sufficiency in pregnancy and lactation (60, 102). EFSA (2014) established an adequate intake (AI) of 70 µg/day for infants 7–11 months and of 90 µg/day for children 1–3 years of age (99) based on the threshold for UIC of 100 µg/day. WHO based their recommendation for infants (90 µg/day) on the intake level needed to achieve positive metabolic balance in a study in Belgian infants (68). The study was conducted at a time when the population in Belgium was iodine deficient (5). WHO has sustained the recommendation of 90 µg/day to ensure sufficient iodine intake in all populations. A new randomised, double-blind, dose-response crossover iodine balance study published in 2016, conducted in 2–5 month old full-term, iodine sufficient infants, showed that iodine balance was achieved at a minimum daily iodine intake of 11 µg/kg, corresponding to 72 µg/day, and proposed a recommended dietary allowance (RDA) of 80 µg/day to maintain adequate iodine status during the first 6 months of life (37). Based on new data and studies from the Nordic and Baltic countries, adjustments of the DRVs should be considered.

### Pregnancy and lactation

During pregnancy and lactation, iodine turnover is increased. In NNR2012, an extra 25 µg/day (175 µg/day) was recommended during pregnancy and an extra 50 µg/day (200) µg/d was recommended during lactation to provide sufficient iodine in the breast milk (103, 104). Evidence from European cohort studies suggests that even mild-to-moderate iodine deficiency during pregnancy may be associated with adverse pregnancy outcomes (87) and subtle impairments in child neurocognitive function (89–94). The results from these studies show that iodine intakes at or above the RI of 150 µg/day for women of childbearing age are associated with the lowest prevalence of adverse health outcomes in mothers and babies. Therefore, ensuring adequate daily iodine in years and months before pregnancy is more important than a large increased in iodine intake after pregnancy has started. If the lactating mother has a sufficient iodine intake, iodine in breast milk will cover the needs of an infant during the first months of life (36). However, the current evidence supports the WHO recommendation that breastfeeding mothers in the Nordic and Baltic countries should use an iodine containing supplement because dietary iodine and current strategies for salt iodisation is not sufficient to meet the requirement of breastfed infants (5, 34).

### Iodine fortification and supplementation

The main challenge regarding iodine nutrition in the Nordic and Baltic countries is that large subgroups of the population are mild-to-moderately iodine deficient, particularly women of childbearing age (64, 73, 75–77, 79, 80) immigrants (105, 106) vegetarians and vegans (31, 98, 107). There are few natural dietary iodine sources, and only Denmark, Finland, and Sweden have implemented mandatory (DK, FI) or voluntary (SE) salt iodisation (18). Universal salt iodisation of all salt is the strategy recommended by WHO (12, 13, 67). This should be accompanied by monitoring of iodine status in groups that are vulnerable to inadequate or excessive intakes (67). In Europe, iodine concentrations in salt vary from 5 μg to 75 μg iodine per gram of table salt. Sweden uses 50 µg/g and Finland uses 25 μg/g (18). In Denmark, the level used to be 13 μg/g, but was increased to 20 μg/g in 2019 in table salt and salt used in industrial bread and bakery items (108).

Results from the Norwegian MoBa study suggest no benefit of iodine supplementation starting in pregnancy, but mixed results with respect to potential negative effects of iodine supplements (7, 89–91). There is not sufficient evidence to recommend initiation of iodine supplementation or fortification during pregnancy in mild-to-moderately iodine deficient populations (23, 28, 30, 32). Recent SRs conclude that initiation of iodine supplementation or fortification during pregnancy is too late to confer benefits (30, 32). Thus, it is important to ensure adequate iodine intake in women of childbearing age, in order for them to enter pregnancy with sufficient thyroidal iodine stores to meet the increased demand. Similarly, available trial data do not show any evidence of beneficial effects of iodine supplementation for preterm infants (26). However, the current evidence supports the WHO recommendation that breastfeeding mothers in the Nordic and Baltic countries should use an iodine containing supplement because dietary iodine and current strategies for salt iodisation is not sufficient to meet the requirement of breastfed infants (5, 34).

### Upper intake levels and toxicity

There is a substantial inter-individual variation with respect to the dose of iodine that can cause adverse effects. This complicates the assessment of an upper safe limit of intake. Persons with normal thyroid function can, in general, tolerate prolonged consumption of iodine up to 1 mg/day (109, 110). EFSA has proposed 600 µg/day of iodine as the safe upper level (UL) for adults (110) (Table 1). The UL is based on elevations in TSH levels after iodine intake and an enhanced response in TSH levels to thyrotropin releasing hormone (TRH) stimulation. These effects are of a biochemical nature and are not associated with any clinically adverse effects. The UL includes an uncertainty factor and is also considered acceptable for pregnant and lactating women. In children, a median UIC ≥ 500 µg/L was found to be associated with increasing TV in children 6–12 years old, but a UIC of 300–500 µg/L was not (111). The authors of that study, however, did not rule out the possibility of adverse effects of a UIC in the range of 300–500 µg/L that were not detected in the study (111).

**Table 1:** Tolerable upper intake levels for iodine for different age groups (EFSA)

Age	UL µg/day^a^
1–3 years	200
4–6 years	250
7–10 years	300
11–14 years	450
15–17 years	500
Adults	600
Pregnant women	600
Lactating women	600

a The ULs for children were derived by adjustment of the adult UL on the basis of metabolic weight (body weight^0.75^) (110). UL = upper level.

## Data gaps for future research

Implementation of universal salt iodisation has been successful in reducing the prevalence of iodine deficiency disorders by increasing iodine intake in vulnerable groups, including school-age children, non-pregnant non-lactating women of reproductive age, pregnant women, lactating women, 0–6 months old infants, and 7–24 months old infants (13). An international, cross-sectional, multicentre study that included 5860 participants from all the above-mentioned population groups and evaluated the effect of universal salt iodisation ~25 mg/kg that covered a high proportion of the total amount of salt consumed. The median UIC increased in all groups, and in infants and young children the median UIC was in the range of 300–400 µg/L, reflecting intakes close to and higher than the existing UL in a substantial proportion of the children (112). However, this was not considered a concern outweighing the benefit of correcting iodine intakes in women of childbearing age. A benefit-risk assessment by the Norwegian Scientific Committee for Food and Environment estimated that moderate iodisation of household salt and salt in bread and bakery products would ensure iodine intakes above the estimated AR for the majority of women of childbearing age, while at the same time result in intakes above the UL for 8–18% of 1–2-year-old children (88). The report concluded that ‘no level of iodisation would benefit all age and gender groups without posing increased risk of harm to others or that the benefits in one population group outweigh the risk in others’. The ULs for infants and young children are extrapolated from adults, and the UL of 200 µg/day in 1–2-year-old children might be too low (5). Future research is needed to re-evaluate the risk of iodine intakes above the current UL of 200 µg/day for 1–2-year-old children versus the benefit of implementing universal salt iodisation to increase iodine intake in women of childbearing age.

More nationally representative data on iodine status in infants and toddlers is warranted. Studies in infants should be aligned with studies in lactating women and include breast milk iodine concentration. Children at particular risk for iodine deficiency include breastfed and weaning infants in countries with no or voluntary salt iodisation at low coverage or fed by mothers on a restrictive diet, and toddlers receiving homemade complementary foods with low iodine content and no added iodised salt (5).

Well-designed randomised controlled trials addressing neurocognitive function of children born in areas of mild-to-moderate iodine deficiency are lacking, including studies assessing the safety of supplementation with iodine during pregnancy. Furthermore, studies that establish optimal concentration range of iodine in breast milk are needed. Several studies have pointed out a discrepancy between what has been considered sufficient iodine intake in breastmilk and the WHO recommendation for breastfed infants (62–64).

The current evidence regarding iodine status and obesity have shown diverging associations, particularly in school children (113). In the Nordic countries, studies in pregnant women show no association between body mass index and UIC (75, 87, 93, 114). Further studies, particularly in children, need to address the role of body mass index as a factor potentially influencing iodine intake and markers of iodine status.

## Conflict of interest and funding

The authors declare no conflict of interest.
